# Increased collagen production in fibroblasts cultured from irradiated skin and effect of TGF **β**_1_– clinical study

**DOI:** 10.1054/bjoc.2000.1321

**Published:** 2000-08-16

**Authors:** M C Illsley, J H Peacock, R J McAnulty, J R Yarnold

**Affiliations:** Institute of Cancer Research, Cotswold Road, Sutton, Surrey, SM2 5NG; University College, University Street, London, WC1E 6JJ; Royal Marsden Hospital, Downs Road, Sutton, Surrey, SM2 5PT

**Keywords:** radiotherapy, fibroblasts, radiation fibrosis, collagen, transforming growth factor beta

## Abstract

Fibrosis in normal tissues is a common and dose-limiting late complication of radiotherapy at many cancer sites, but its pathogenesis is poorly understood. We undertook a controlled study of the effect of irradiation on the collagen production of fibroblasts cultured from skin biopsies taken from patients undergoing radiotherapy treatment. Eight weeks after a single 8 Gy fraction using 300 kV X-rays, five patients treated at the Royal Marsden Hospital underwent biopsy of the irradiated site and of the contralateral, unirradiated body site. Fibroblasts from irradiated and control, unirradiated sites were cultured in vitro, and collagen production rates were measured during a 48-hour incubation under standardized conditions and in the presence and absence of transforming growth factor β_1_(TGF β_1_), 1 ng/ml, using HPLC. Collagen production was elevated in cells cultured from irradiated skin; median collagen production rates 61.16 pmoles hydroxyproline/10^5^cells/hour in irradiated cells, 39.78 pmoles hydroxyproline/10^5^cells/hour in unirradiated cells, *P* = 0.016 (Mann–Whitney U-test). In fibroblasts from unirradiated sites, collagen production rates were increased by the addition of TGF β_1_; however, in three of the cell lines cultured from irradiated sites this effect of TGF β_1_ on collagen production was not observed. © 2000 Cancer Research Campaign


*British Journal of Cancer* (2000) **83**(5), 650–654
© 2000 Cancer Research Campaign
doi: 10.1054/ bjoc.2000.1321, available online at http://www.idealibrary.com on IDEAL®

### Increased collagen production in fibroblasts cultured from irradiated skin and effect of TGF $\beta_1$ – clinical study

**MC Illsley[1], JH Peacock[1], RJ McAnulty[2] and JR Yarnold[3]**

[1]Institute of Cancer Research, Cotswold Road, Sutton, Surrey SM2 5NG; [2]University College, University Street, London WC1E 6JJ; [3]Royal Marsden Hospital, Downs Road, Sutton, Surrey SM2 5PT

**Summary** Fibrosis in normal tissues is a common and dose-limiting late complication of radiotherapy at many cancer sites, but its pathogenesis is poorly understood. We undertook a controlled study of the effect of irradiation on the collagen production of fibroblasts cultured from skin biopsies taken from patients undergoing radiotherapy treatment. Eight weeks after a single 8 Gy fraction using 300 kV X-rays, five patients treated at the Royal Marsden Hospital underwent biopsy of the irradiated site and of the contralateral, unirradiated body site. Fibroblasts from irradiated and control, unirradiated sites were cultured in vitro, and collagen production rates were measured during a 48-hour incubation under standardized conditions and in the presence and absence of transforming growth factor $\beta_1$ (TGF $\beta_1$), 1 ng/ml, using HPLC. Collagen production was elevated in cells cultured from irradiated skin; median collagen production rates 61.16 pmoles hydroxyproline/$10^5$ cells/hour in irradiated cells, 39.78 pmoles hydroxyproline/$10^5$ cells/hour in unirradiated cells, $P = 0.016$ (Mann–Whitney U-test). In fibroblasts from unirradiated sites, collagen production rates were increased by the addition of TGF $\beta_1$; however, in three of the cell lines cultured from irradiated sites this effect of TGF $\beta_1$ on collagen production was not observed. © 2000 Cancer Research Campaign

**Keywords:** radiotherapy; fibroblasts; radiation fibrosis; collagen; transforming growth factor beta

Radiation fibrosis is a common and dose-limiting late complication of radiotherapy treatment for many cancers, and is prominent in skin, subcutaneous tissue, lung, muscle, pericardium, heart, and the submucosa of gastrointestinal and urinary tracts (Rubin and Cassarrett, 1968). Changes described in the pathological literature include patchy replacement of parenchyma by dense, irregular collagen fibres, with resultant encasement and sometimes compression of adjacent structures. Involved tissue becomes hardened, rigid and less compliant than normal, often with accompanying progressive tissue shrinkage, resulting in impaired function (Rubin and Cassarrett, 1968).

Fibrosis is believed to be due to alterations in extracellular matrix, mainly collagen, deposition by soft tissue fibroblasts, under the influence of fibrogenic cytokines, including isoforms of transforming growth factor beta (TGF β). Induction of TGF β synthesis and alterations in parameters of collagen synthesis have been reported in animal studies as early events after irradiation (Barcellos-Hoff 1993; Finkelstein et al, 1994). Increased local TGF β synthesis after irradiation in humans has been demonstrated by Canney and Dean (1990), who detected the cytokine in tissue sections taken at surgery from six patients undergoing resection of large bowel carcinomas following fractionated pre-operative radiotherapy.

It has been well documented that irradiation causes impairment of the clonogenic potential of fibroblasts, and may also result in changes in their differentiation status (Rodemann and Bamberg, 1995). However, the effect of irradiation on fibroblast cellular function, including collagen synthesis, has been little studied to date in the clinical setting.

This controlled clinical study examines the effect of irradiation in vivo on fibroblast collagen synthesis. Fibroblasts cultured from biopsies taken from irradiated and unirradiated (control) skin are compared in terms of their rates of collagen synthesis in vitro and fibrogenic response to added TGF $\beta_1$ under standardized conditions. Collagen production is measured using an ultrasensitive HPLC technique capable of detecting picomolar concentrations of hydroxyproline, an imino acid highly selective for collagen (Campa et al 1990).

Since TGF β is known to affect the proliferation and cell cycle characteristics of fibroblasts cultured in vitro (Zhang and Jacobberger, 1996), and in order to calculate collagen synthesis rates per cell, flow cytometric analysis of DNA content and cell counts are performed in parallel with the collagen production assays.

*Received 4 January 2000*
*Accepted 5 May 2000*

*Correspondence to:* MC Illsley, St Lukes Cancer Centre, Royal Surrey County Hospital, Egerton Road, Guildford GU2 5XX, Surrey, UK

### MATERIALS AND METHODS

### Patients and biopsies

Patients were prospectively selected from those undergoing radiotherapy using 300 kV X-rays at the Royal Marsden Hospital, Sutton, Surrey, and were recruited for the study prior to their irradiation. In all cases the skin biopsied was clinically and histologically unaffected by malignancy. The purpose of treatment for these patients was the palliation of pain from metastatic cancer affecting underlying bone. The dose of irradiation was 8 Gy single fraction prescribed at the skin surface, to the centre of a field measuring

**Table 1** Clinical details of patients recruited

| Study no | Age (y) | Sex | Site of primary | Site of radiotherapy |
|---|---|---|---|---|
| 1 | 77 | Male | Prostate | Right scapula |
| 2 | 66 | Male | Colon | Right posterior ribs |
| 3 | 59 | Female | Lung | Right scapula |
| 4 | 80 | Female | Breast | Right posterior ribs |
| 5 | 75 | Female | Breast | Right posterior ribs |

between $8 \times 8$ and $10 \times 12$ cm. This treatment technique delivers a high dose of radiotherapy to an area of tissue which is not involved by cancer.

Eight weeks after irradiation, skin biopsies were taken from the centre of the irradiated area and control biopsies from an area of skin on the contralateral, unirradiated body site. Biopsies were taken using a 3 mm punch under 2% lignocaine local anaesthesia. It was found in all cases that, at the time of biopsy, the irradiated site was delineated by a faint zone of erythema, enabling the biopsies to be taken with confidence from the centre of the treatment field.

Written informed consent was obtained from all patients prior to inclusion, and the study protocol was approved by the Ethics Committee of the Royal Marsden Hospital. Clinical details of the five patients recruited are given in Table 1. None of the patients were receiving chemotherapy or other drugs known to enhance the effect of radiotherapy.

### Cell culture

Biopsies were transported to the laboratory in Dulbecco's Modified Eagle's Medium (DMEM) with 10% fetal calf serum (FCS), cut into small fragments and immediately explanted into 12.5 $cm^2$ culture flasks (Falcon) in 0.5–1 ml DMEM containing 10% FCS, 10 mM Hepes with penicillin, 50 U/ml and streptomycin, 50 µg/ml. One flask was set up per 3 mm punch biopsy, and each biopsy was handled separately.

Explanted biopsies were left undisturbed for ten days, at which time the culture medium was removed along with any pieces of tissue unattached to the bottom of the flask. When fibroblasts reached confluence or became crowded in one area of the flask, they were passaged up into a single 25 $cm^2$ flask (P1), and subsequently to a single 80 $cm^2$ flask (P2). Cells in four $T_{80}$ flasks (passage 3) were allowed ten days to reach confluence before passaging and seeding into 12-well plates at a density of $10^5$/ml in DMEM + 10% FCS, 1 ml/well. After three days the medium was changed. One week after plating, cells were checked under the microscope for visual confluence, and the medium was removed and replaced with DMEM containing 0.5% FCS, 50 µg/ml ascorbic acid, and 0.2 mM L-proline (pre-incubation medium). Twenty-four hours later, at time $T_0$, the experimental incubation was commenced by removal of preincubation medium and its replacement by incubation medium (fresh pre-incubation medium with or without TGF $\beta_1$ (R and D systems, UK) at a concentration of 1 ng/ml). The experiment was terminated 48 hours later.

Experiments were standardised in order to enable valid comparison of results. Medium used for all experiments was from the same batch, and kept at 4°C with no additions in preparation for experiments. A single bottle of fetal calf serum and a single vial of TGF $\beta_1$ were aliquoted into volumes suitable for single experiments and frozen at – 70°C until use. All experiments described, apart from the preparatory experiment demonstrating the kinetics of collagen synthesis, were performed with these reagents and media. The timing of cell passaging into multiwells in preparation for experiments was always the same, in order to, as far as possible, ensure that comparison of parameters of cell function was valid. For each pair of cell lines (prepared from biopsies of irradiated, and unirradiated, skin from one patient) collagen production experiments were done simultaneously.

### Measurement of collagen production

The method used was based on previously described protocols (McAnulty et al, 1995). This exploits the reactivity of 7-chloro-4-nitro-benzo-2-oxa-1,3-diazole (NBD-C1) with hydroxyproline to yield a reaction product exhibiting strong light absorbence at 450–550 nm, which can be sensitively and accurately quantified by high performance liquid chromatography (HPLC).

At the end of the experiment, fibroblast cell layers were scraped into the experimental medium, which was collected in plastic conical tubes. Well contents were washed out with a further 1 ml PBS, and the washings added to the initial samples. Proteins in the samples were precipitated by the addition of 4 ml ice-cold absolute ethanol, to a final ethanol concentration of 67% (v/v) and the samples kept at 4°C overnight. The protein-bound fraction was extracted from the sample by filtration (0.45 µm, Millipore), and filters bearing the protein-bound precipitates hydrolysed in 2 ml HCl for 16 hours at 110°C. Samples were decolourized by the addition of 7 mg charcoal, filtered (0.65 µm, Millipore) and a 100 µl aliquot of each sample lyophilised in preparation for derivitization.

Samples were re-dissolved in 100 µl deionized water, and 100 µl 0.4 M potassium tetraborate, pH 9.5 added, followed by the addition of 100 µl 12 mM NBD-Cl in methanol, and the sample thoroughly mixed, then incubated at 37°C for 20 minutes in the dark. The derivitization reaction was stopped by the addition of 50 µl 1.5 M HCl followed by 150 µl 167 mM sodium acetate, pH 6.4 in 26% (v/v) acetonitrile. Samples were filtered (0.22 µm) and a 100 µl aliquot run on the HPLC column. A characteristic peak representing hydroxyproline was observed at 5.5–7.0 minutes. Hydroxyproline content was determined by comparing peak areas of samples from the chromatogram with those generated from standard samples of hydroxyproline derivitized and run on the same day. Collagen production rates were expressed as pmol hydroxyproline/$10^5$ cells/hour.

### Cell counts

Fibroblasts from duplicate wells were brought into suspension by incubation for 15 minutes in 500 µl trypsin. The cells were thoroughly disaggregated and mixed using a Gilson pipette tip, and 200 µl of the cell suspension added to 4.8 ml of PBS in preparation for cell counting using a Coulter counter.

### Flow cytometry

The technique used was that of Ormerod (1990) for flow cytometric analysis of cellular DNA content. The remainder of the disaggregated cell suspension was centrifuged with 2 ml medium containing 10% FCS for 10 min at 800 rpm, the resulting cell

© 2000 Cancer Research Campaign

**Table 2** Cell counts, values expressed as count/well × $10^{-5}$

| Patient study no | Without TGF $\beta_1$ Unirradiated | Irradiated | With TGF $\beta_1$ Unirradiated | Irradiated |
|---|---|---|---|---|
| 1 | 2.27 | 1.64 | 2.26 | 1.83 |
| 2 | 2.08 | 1.43 | 2.05 | 1.35 |
| 3 | 2.03 | 0.74 | 1.45 | 0.68 |
| 4 | 2.43 | 1.47 | 2.49 | 1.51 |
| 5 | 1.61 | 1.58 | 1.62 | 1.52 |

Values shown are the means of four estimations; duplicate counts from duplicate wells at the end of the 48 hour experimental incubation pellet washed in 4 ml PBS and re-spun, then fixed in 500 μl ice-cold absolute ethanol. After further washing in PBS, RNA removal was performed by the addition of RNase (1 mg/ml) and the cells then incubated at 37°C for 30 minutes in the presence of propidium iodide (400 μg/ml) and analysed in an OrthoCytofluorograph 50 H flow cytometer equipped with a 50 mW Lexel argon-ion laser. The cell cycle parameters were obtained using interfaced Ortho 2150 computer software and figures quoted are taken directly from this system.

### RESULTS

### Cell counts

Table 2 shows cell counts obtained from duplicate wells at the end of the 48 hour experimental incubation in the presence or absence of TGF $\beta_1$, 1 ng/ml, expressed as cell count per well × $10^{-5}$. Counts are given as the mean of four estimations; duplicate counts were performed on duplicate wells.

This shows cell counts in fibroblasts from irradiated sites to be lower than those from unirradiated sites of the same patient in these culture conditions, mean cell counts (× $10^{-5}$) 2.08 (SEM 0.14) for unirradiated cells, 1.37 (SEM 0.16) for irradiated cells, $P$ = 0.027, student's two-tailed t-test. The presence or absence of TGF $\beta_1$ can be seen to have no effect on cell counts obtained.

### Flow cytometry

Results of flow cytometry are given for unirradiated fibroblasts in Table 3a and for irradiated fibroblasts in Table 3b. The data indicates that during a 48-hour incubation, in the presence or absence of TGF $\beta_1$, there was no alteration in the cell cycle characteristics of these cells, the majority remaining in $G_1/G_0$ for the duration of the experiment.

### Collagen production

The kinetics of collagen production are shown in Figure 1. This experiment was performed using cultured dermal fibroblasts obtained from a mammoplasty specimen, and demonstrates collagen production to be linear during a 48-hour incubation under the experimental conditions described.

Rates of collagen production for unirradiated and irradiated cells from patients biopsied as part of the current study in the presence and absence of TGF $\beta_1$, 1 ng/ml, are shown in Figure 2.

The figure shows collagen production for control, unirradiated dermal fibroblasts from the five patients studied to be between 29.80 and 82.84 pmoles OHpro/$10^5$ cells/hour, median

**Table 3a** Cell cycle distribution of fibroblasts in culture at $T_0$, and at end of 48 hour experimental incubation, in presence and absence of TGF $\beta_1$, 1 ng/ml, for unirradiated cells

| | $T_0$ | Without TGF $\beta_1$ | With TGF $\beta_1$ |
|---|---|---|---|
| % $G_1$ | 89.91 +/– 1.68 | 91.25 +/– 1.37 | 88.36 +/– 1.55 |
| % S | 4.69 +/– 0.35 | 3.49 +/– 0.63 | 4.49 +/– 0.80 |
| % $G_2$ | 5.35 +/– 1.56 | 5.17 +/– 1.32 | 7.18 +/– 1.20 |

Values represent percentage of cell population in each phase of the cell cycle and are given as the means of duplicate estimates for patients 1–5; that is, means of ten estimates +/– 1 SEM

**Table 3b** Cell cycle distribution of fibroblasts in culture at $T_0$, and at end of 48 hour experimental incubation, in presence and absence of TGF $\beta_1$, 1 ng/ml, for irradiated cells

| | $T_0$ | Without TGF $\beta_1$ | With TGF $\beta_1$ |
|---|---|---|---|
| % $G_1$ | 89.39 +/– 1.68 | 89.51 +/– 1.17 | 87.08 +/– 0.69 |
| % S | 5.38 +/– 0.68 | 5.32 +/– 0.66 | 6.39 +/– 0.89 |
| % $G_2$ | 5.19 +/– 1.05 | 5.15 +/– 1.16 | 6.54 +/– 1.03 |

Values represent percentage of cell population in each phase of the cell cycle and are given as the means of duplicate estimates for patients 1–5; that is, means of ten estimates +/– 1 SEM

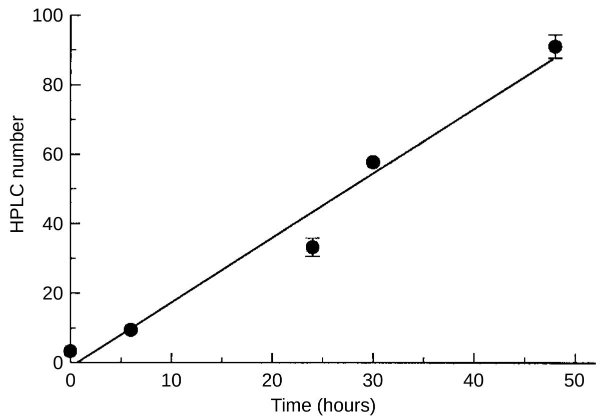

**Figure 1** Kinetics of in vitro collagen production in cultured dermal fibroblasts. Values represent mean +/–1 SEM of 4–6 wells

39.78 pmoles hydroxyproline (OHpro)/$10^5$ cells/hour. Fibroblasts cultured from all five patients studied demonstrated increased collagen production in cells from irradiated areas compared with controls, mean collagen production in irradiated cell lines 61.16 pmoles OHpro/$10^5$ cells/hour, $P$ = 0.016, Mann-Whitney U test. It can be seen from Figure 2 that errors within each set of 4–6 wells analysed were small, indicating good assay reproducibility.

Addition of TGF $\beta_1$, at a concentration of 1 ng/ml, to the culture medium increased the rate of collagen production in all control cell lines, by a factor of between 1.31 and 1.91, consistent with existing data (McAnulty et al, 1991). However, it can be seen from Figure 2 that the effect of added TGF $\beta_1$ on fibroblasts from irradiated areas is not consistent; fibroblasts from patients 1, 4 and 5 did not respond to TGF $\beta_1$, whereas those from patients 2 and 3 showed a similar response to the control cells.

© 2000 Cancer Research Campaign

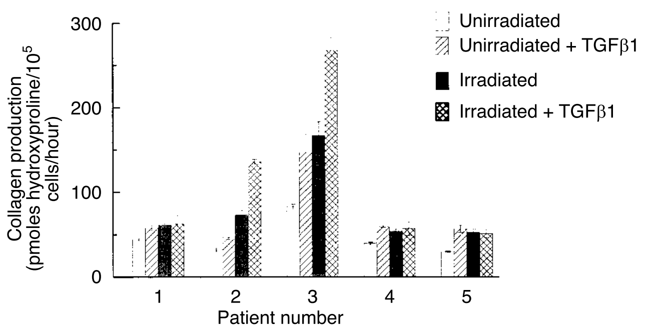

**Figure 2** Collagen production by control and irradiated cells in presence and absence of TGF $\beta_1$, 1 ng/ml. Values represent mean +/–1 SEM of 4–6 wells

### DISCUSSION

This study demonstrates that fibroblasts derived from irradiated areas from patients biopsied soon after radiotherapy can be established and maintained in culture and retain their ability to produce collagen; indeed, their collagen production is enhanced when compared to that in unirradiated cells from the same individuals cultured under equivalent conditions. This is the first demonstration of such a phenomenon to date in a clinical study. It was observed consistently in cell lines from all 5 patients studied, though the magnitude of the difference was variable. Because of the standardization of experimental conditions in this study, the observed elevation of collagen synthesis rates in irradiated cells when compared to controls cannot be explained on the basis of differences in variables known potentially to affect the functional characteristics of fibroblasts in culture, such as passage number (E1 Nabout et al, 1989) or local cytokine environment (Moulin et al, 1997).

Our demonstration of increased collagen production rates in cells cultured from areas irradiated in vivo is consistent with the finding of Kolb et al (1998) of a radiation dose-dependent increase in collagen production (as measured by an ELISA estimation of the concentration of collagen I propeptide) by human lung fibroblasts irradiated in vitro. The demonstration of increased concentrations of collagen propeptides in interstitial fluid from the skin of patients with subcutaneous fibrosis following breast radiotherapy (Autio et al, 1998) indicates that this phenomenon is also likely to be relevant in the clinical situation. Although the radiation dose employed in the current study, 8 Gy single fraction, would not be expected to cause significant clinical radiation fibrosis, it is likely that elevated collagen synthesis by irradiated fibroblasts, as demonstrated in this study, contributes to the generation of fibrosis seen after higher doses. Experiments such as these would not be possible on fibroblasts irradiated to a high dose, because of the impairment of clonogenic potential caused, which makes these cells difficult to maintain in culture. It would not, therefore be possible to generate sufficient of these cells in culture to perform quantitative experiments.

The current study was performed on fibroblasts at passage 4, which was the minimum passage number at which sufficient cells were available to perform the experiments. Experiments were done at the earliest possible passage after biopsy in order to minimize effects of cell selection during serial passage. However, the elevation of collagen production rates at passage 4 has interesting implications in terms of the pathogenesis of radiation fibrosis, suggesting that irradiation in vivo induces an altered cell phenotype which is subsequently stable in cell culture. This would be consistent with the hypothesis that these cells have undergone, as a result of irradiation, a stable phenotypic change, a component of which is increased or upregulated collagen synthesis. This is consistent with the observations of Rodemann and Bamberg (1995) that irradiation in vitro induces so-called 'terminal differentiation' of fibroblasts, and also with our observation of lower cell numbers in wells containing irradiated cells, implying diminished growth potential, and our informal observation that cells from irradiated sites tended to be larger in size than control cells (data not shown).

Addition of TGF $\beta_1$, at a concentration of 1 ng/ml, to the culture medium increased the rate of collagen production in all control cell lines, by a factor of between 1.31 and 1.91, which is consistent with existing data from this laboratory (McAnulty et al, 1991). Since the results of flow cytometry show that there was no change in cell cycle parameters during the time course of the experiment, this effect cannot be explained on the basis of an effect of the cytokine on cellular proliferation. Interestingly, in cell lines from three of the patients studied, the response to TGF $\beta_1$, which was present in unirradiated cells, was no longer present in irradiated cells from the same patient. However, in irradiated cell lines from the two remaining patients, addition of the cytokine to the culture medium resulted in an increase in collagen production. The reason for this apparent disparity in response is currently unclear.

Alterations in in vitro cellular responses to TGFβ have been noted in scleroderma, a disease of unknown aetiology characterized by dermal fibrosis. Fibroblasts obtained from the affected skin of patients with this disease display elevated collagen synthesis when cultured in vitro; however, experimental data on their fibrogenic responses to TGFβ is conflicting, and the role of TGFβ in the pathogenesis of the disease in vivo is unclear (for review see Cotton et al, 1998). The functional characteristics of fibroblasts derived from irradiated areas are poorly understood, partly because their diminished clonogenic potential as a result of irradiation makes them difficult to culture in vitro. Therefore, unlike fibroblasts from other fibrotic conditions, such as scleroderma, whose collagen synthesis and fibrogenic responses to cytokines in vitro have been closely studied, little data exists on the alterations in cellular function induced by irradiation. This study is, we believe, the first to demonstrate that fibroblasts from patients can be cultured and studied in vitro shortly after radiotherapy treatment, and display enhanced collagen synthesis which is likely to be relevant to the mechanism of radiation fibrosis in the clinical situation. It is important to emphasize, however, the complexity of the dynamics of the extracellular matrix, which is recognised to undergo constant turnover, with collagen degradation processes, not assayed in the current study, also thought to be of importance to the generation of extracellular matrix remodelling and tissue fibrosis. It is hoped that further study of changes in parameters relevant to fibrosis following radiotherapy will clarify more fully the pathological processes occurring.

### ACKNOWLEDGEMENTS

We gratefully acknowledge the assistance of Jane Regan in patient recruitment, consultants at the Royal Marsden Hospital, Sutton for allowing inclusion of their patients in this study; also Caroline

*© 2000 Cancer Research Campaign*

Jackson for assistance with statistics and Jenny Titley for assistance with flow cytometry. MI was funded by a CRC Clinical Research Fellowship.

### REFERENCES

Autio P, Saarto T, Tenhunen M, Elomaa I Risteli J and Lahtinen T (1998) Demonstration of increased collagen synthesis in irradiated human skin in vivo. *Br J Cancer* **77(12)**: 2331–2335

Barcellos-Hoff MH (1993) Radiation-induced transforming growth factor β and subsequent extracellular matrix reorganization in murine mammary gland. *Cancer Res* **53**: 3880–3886

Campa JS, McAnulty RJ and Laurent GJ (1990) Application of high pressure liquid chromatography to studies of collagen production by isolated cells in culture. *Anal Biochem* **186**: 257–263

Canney PA and Dean S (1990) Transforming growth factor beta: a promoter of late connective tissue injury following radiotherapy? *Br J Radiol* **63**: 620–623

Cotton SA, Herrick AL, Jayson MV and Freemont of AJ (1998) TGF β – a role in systemic sclerosis? *J Pathol* **184**: 4–6

El Nabout R, Martin M, Remy J, Kern P, Robert L and Lafuma C (1989) Collagen synthesis and deposition in cultured fibroblasts from subcutaneous radiation-induced fibrosis. Modification as a function of cell aging. *Matrix Biol* **9**: 411–420

Finkelstein JN, Johnston CJ, Baggs R and Rubin P (1994) Early alterations in extracellular matrix and transforming growth factor β gene expression in mouse lung indicative of late radiation fibrosis. *Int J Radiat Oncol Biol Phys* **28 (3)**: 621–631

Kolb M, Wruck R, Rollin J, Kirschner J, Willner J and Schmidt M (1998) Lung fibroblasts produce high amounts of fibronectin after irradiation. *Am J Respir Crit Care Med* **157**: A 846

McAnulty RJ, Campa JS, Cambrey AD and Laurent GJ (1991) The effect of transforming growth factor β on rates of procollagen synthesis and degradation in vitro. *Biochemica et Biophysica Acta* **1091**: 231–235

McAnulty RJ, Chambers RC and Laurent GJ (1995) Regulation of fibroblast procollagen production. *Biochem J* **307**: 63–68

Moulin V, Auger F, O'Connor-McCourt M and Germain L (1997) Fetal and postnatal sera differentially modulate human dermal fibrobast phenotypic and functional features in vitro. *J Cell Physiol* **171**: 1–10

Ormerod MG (1990) (ed) *Flow cytometry. A practical approach*. IRL Press (Oxford University Press)

Randall K and Coggle JE (1996) Long-term expression of transforming growth factor TGF $\beta_1$ in mouse skin after localized β-irradiation. *Int J Radiat Biol* **70(3)**: 351–360

Rodemann HP and Bamberg M (1995) Cellular basis of radiation-induced fibrosis. *Radiother Oncol* **35**: 83–90

Rubin P and Cassarett GW (1968) *Clinical Radiation Pathology*. Philadelphia: W.B. Saunders Company

Rudolph R, Vande-Berg J, Scheider JA, Fisher JC and Poolman WL (1988) Slowed growth of cultured fibroblasts from human radiation wounds. *Plast Reconstr Surg* **82(4)**: 669–677

Zhang D and Jacobberger JW (1996) Perturbation of cell cycle by TGF $\beta_1$. *Cell Prolif* **29**: 289–307

 *© 2000 Cancer Research Campaign*
